# Are Gamers Prone to eThrombosis during Long Gaming Sessions?

**DOI:** 10.3390/life14040525

**Published:** 2024-04-18

**Authors:** Kasper B. Krarup, Henrik B. Krarup, Morten Mørk, Søren Lundbye-Christensen, Aase Handberg, Hien T. T. Nguyen, Inge S. Pedersen, Søren R. Kristensen

**Affiliations:** 1 Department of Geriatrics, Aalborg University Hospital, 9000 Aalborg, Denmark; kasper.krarup@rn.dk; 2 Sports Medicine Center, Region Hospital North Jutland, 9900 Frederikshavn, Denmark; 3 Department of Molecular Diagnostics, Aalborg University Hospital, 9000 Aalborg, Denmark; h.krarup@rn.dk (H.B.K.); hien@rn.dk (H.T.T.N.); isp@rn.dk (I.S.P.); 4 Department of Clinical Medicine, Aalborg University, 9000 Aalborg, Denmark; solc@rn.dk (S.L.-C.);; 5 Department of Clinical Biochemistry, Aalborg University Hospital, 9000 Aalborg, Denmark; morten.moerk@rn.dk

**Keywords:** eThrombosis, LAN party, thrombin generation, coagulation activation, TAT, F1 + 2, D-dimer

## Abstract

During the last two decades, several cases of venous thrombosis (VTE) after a prolonged period at a computer have been described, denominated as “eThrombosis”. Video gaming on a computer has become very popular and can be a social activity where several players gather to play against each other or in a virtual environment for several days (“LAN (i.e., **L**ocal **A**rea **N**etwork) parties”) where the participants are sedentary and consuming calorie-rich food items. The aim of this study was to investigate potential coagulation activation during a 42 h LAN party. Nine male gamers volunteered for the LAN party. Citrated blood was sampled before and every 6 h, and plasma was analyzed for thrombin generation, thrombin–antithrombin complexes (TAT), prothrombin fragment 1 + 2 (F1 + 2), and D-dimer. Thrombin generation increased slightly but not significantly during the LAN party, whereas the coagulation activation markers were unchanged. These results do not indicate that the coagulation system is activated significantly during 42 h of gaming with minimal physical activity. Although increased activity cannot be excluded, it does not directly indicate a risk of VTE in general.

## 1. Introduction

Venous thromboembolism (VTE) comprising deep venous thrombosis and pulmonary emboli is a multicausal disease [1] with several risk factors. This encompasses genetic risk factors as gain-of function mutations, e.g., *Factor V Leiden* and *Factor II G20210A*; or the lack of one or dysfunctional forms of the coagulation inhibitors, antithrombin and Protein C and S; and provoking factors such as surgery, immobilization, estrogen use, pregnancy, etc. The provoking factor immobilization may lead to stasis, one of the factors of Virchow’s triad, which may lead to a dysfunctional and procoagulant endothelium and, subsequently, hypercoagulation. The effect of immobilization is difficult to quantify. One attempt to estimate this effect was carried out by Ocak et al. in a group of patients with VTE; they estimated that immobilization (defined as being bedridden at home for at least 4 days, being hospitalized, or having surgery within 3 months) caused a six-fold increased risk of VTE in patients without a major disease and a 10-fold increased risk in patients with a major disease [2]. Thus, immobilization can be a substantial risk factor for VTE.

It is well known that long-haul flights or long bus journeys increase the risk of VTE. This is, at least partly, due to immobility, where stasis in the legs predisposes one to thrombosis [3]. Analogous to this kind of seated immobilization, it has been described that sedentary work at a desktop computer may predispose one to VTE. In 2003, Beasley et al. described a patient who developed venous thrombosis (VTE) while working at a computer regularly for 12 h per day, sometimes up to 18 h. Typically, he was sedentary for several hours—not infrequently 6 h—at the computer without a break [4]. In the absence of other provoking risk factors, this prolonged inactive sitting was considered the provoking factor, and the authors proposed the name “eThrombosis”. At the same time, another publication by Ng et al. described a similar case, i.e., deep venous thrombosis in a 12-year-old boy who had been playing on a games console for four consecutive hours [5]. In the following years, several cases of otherwise healthy persons, even children, who developed VTE after prolonged computer-related activity were published [6,7,8,9]. In 2018, Lippi et al. published a review paper on eThrombosis, describing previous cases and the possible physiopathology behind it [10]. During the COVID-19 pandemic, it was stated in some correspondences that the increased use of online meetings and increased homework might increase the risk of eThromboses [11,12]. Very recently, another case of the unexpected death of an information technology professional because of a pulmonary embolism was published; he had been working at home for a year [13]. 

The mechanism of immobility and stasis is poorly understood, and the risk factors described by Virchow’s triad, blood stasis, endothelial damage, and hypercoagulability, are probably inter-related [14]. Stasis, i.e., a reduction in the shear stress and rate of the blood, may result in several changes, including hypoxia and the inflammation of endothelial cells and the activation of leucocytes. Subsequently, this may activate the coagulation system by various mechanisms [15]. However, the potential activation of the coagulation system during prolonged computer work has not been investigated. 

Video gaming on a computer or games console has become very popular and can be a social activity where several players are gathered to play against each other or are in a virtual environment on the internet. LAN (Local Area Network) parties are casual or competitive events where a number of players participate, bringing along their computers which are linked to a network or via the internet, and they play for several days [16,17]. During these days, the participants play most of the time with minimum sleep, or even no sleep, and they usually eat and drink unhealthy items such as pizza, burgers, chips, and other snacks, and lots of soda and soft drinks [16,17]. We recently arranged a party like this from Friday to Sunday where the participants only slept for about 6 h and consumed calorie-rich food items [16]. Since prolonged sedentary activity at a computer may be a risk factor for VTE, this observational, exploratory study aimed to investigate the effect of the 42 h long LAN party with minimal physical activity on coagulation activity and activation. To describe the activity of the coagulation system, we measured changes in thrombin generation, describing the potential activity of the entire coagulation system, whereas the actual activation of the coagulation system was quantitated by the following coagulation markers: the thrombin–antithrombin complex (TAT), prothrombin fragment 1 + 2 (F1 + 2), and D-dimer. Our hypothesis is that the activity and/or the potential of the coagulation system may be increased during a prolonged sedentary period. The results showed a small but nonsignificant increase in the coagulation potential, but the markers of coagulation activity did not increase.

## 2. Materials and Methods

### 2.1. Participants

This pilot study has been described before [16]. In brief, nine male gamers volunteered to participate in a LAN party after the announcement of the study on several gaming-related message boards. The inclusion criteria were healthy male adults (>18 years old), and the exclusion criteria were anemia, hypertension, and diabetes mellitus. They arrived on a Friday afternoon, and the gaming period consisted of two 18 h sessions from 6 p.m. to noon the next day, interrupted by 4–5 h sleep on Saturday afternoon before gaming resumed at 6 p.m. and ended on Sunday at noon. The study was approved by the local Ethics Committee (N20180011).

### 2.2. Blood Samples

Blood was sampled right before the start at 6 p.m. Friday and again every 6 h until Sunday at noon, and finally, sampling on the following Friday. Venous blood was collected using a 21-gauge needle into 3.2% (*w*/*v*) trisodium citrate (Vacutainer, Becton Dickinson, Plymouth, UK)) and centrifuged at room temperature at 2500× *g* for 15 min. The supernatant to 1 cm above the buffy coat was recentrifuged for another 15 min at 2500× *g*, and plasma to 1 cm above the pellet was stored at −80 °C within one hour until analysis.

### 2.3. Analyses 

Thrombin generation (TG) was performed using a calibrated automated thrombogram on a FluoStar Optima device (BMG Labtech, Ortenberg, Germany) using Thrombinoscope Software (Ascent Software 2.6) (Thrombinoscope BV, Maastricht, The Netherlands) according to the manufacturer’s instructions: 80 µL of each sample was mixed with 20 µL of trigger reagent and 20 µL of buffer containing a fluorogenic substrate (Z-Gly-Gly-Arg-AMC) and CaCl_2_ (FluCa kit). As the trigger reagent, PPP-Reagent Low containing 1 pM tissue factor (TF) and 4 µM of phospholipids (PLs) was used, and each sample was calibrated against a calibrator containing a fixed amount of thrombin–α2-macroglobulin complex (all reagents were from Diagnostica Stago, Asnière-sur-Seine, France). Dedicated Thrombinoscope software (Thrombinoscope BV) was used to calculate Lagtime (the time from initiation to start of thrombin generation); endogenous thrombin potential (ETP), i.e., all thrombin formed during the process; the peak (maximal concentration of thrombin formed); and time to peak (ttPeak), i.e., the time from the initiation to the peak. 

ELISA kits for TAT (Enzygnost TAT Micro Kit) and F1 + 2 (Enzygnost F1 + 2 (monoclonal)) were from Siemens Healthineers (Marburg, Germany). The reference interval for TAT was <4.2 µg/L, and for F1 + 2, it was 69–229 pmol/L. The ELISA kit for D-dimer (Imuclone D-Dimer ELISA) was from Biomedica Diagnostics (Windsor, ON, Canada). The reference interval was <400 µg/L. The ELISA tests were performed as described in the inserts on BioTek ELx808 and BioTek ELx50 devices (BioTek Instruments Inc. (Winooski, VT, USA)). 

Routine clinical biochemical analyses were performed on a Cobas 8000 Modular Analyzer (Roche Applied Science, Penzberg, Germany), and hematology analyses were performed on Sysmex CS-2100i (Sysmex Europe GmbH, Norderstedt, Germany).

### 2.4. Statistics

The time courses of TG and coagulation parameters were analyzed by repeated-measures ANOVA regression, taking into account the within-gamer correlation. The figures show the values for each gamer during the gaming session and the estimated mean of all nine participants. The trend over gaming time was assessed by fitting a linear trend to the repeated-measures ANOVA model. For this calculation, only the 8 samples within the weekend were included, but the sample from the Friday one week later is also included in the figures for illustration.

## 3. Results

The baseline characteristics of the nine gamers are listed in Supplementary Table S1. They had a mean age of 25.8 (±2.6 (SD)) years, and the mean BMI was 24.8 (±2.9) kg/m^2^. Three were overweight (BMI 26.6–29.0 kg/m^2^), and none were obese. During the two 18 h sessions (interrupted by the 6 h sleeping break), they had a total energy intake of 8005 (±438) kCal (mainly fat and carbohydrates) and a total liquid intake of 6625 (±801) mL containing 1844 (±365) kcal. They were healthy young people (all being in employment or studying) without any known diseases and were physically fit. All routine clinical biochemical analyses (electrolytes, metabolic, and kidney and liver function tests) and hematology analyses (hemoglobin, leucocytes, and platelets) were within reference intervals. All the participants were experienced gamers who were gaming 1–5 h per day (median 3.9 h), and they frequently participated in long gaming sessions online. The participants decided themselves which games they wanted to play, and several games were chosen during the period, but the most-played game was *Counter-Strike: Global Offensive*.

**Figure 1:**
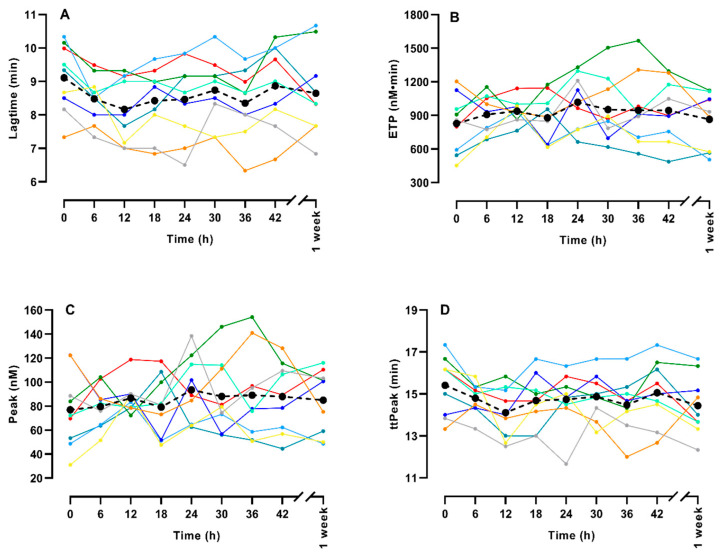
Thrombin generation during a LAN party for 9 healthy gamers. The figures show Lagtime (**A**), Endogenous thrombin potential (ETP) (**B**), time to peak (ttPeak, (**C**)), and peak (**D**). Each colored line depicts one of the gamers, whereas the black dot (●) and the dashed black line depicts the mean of the nine gamers. Please note that the values after one week are not included in the trend analysis—they are only included in the figure for comparison.

Figure 1 shows the thrombin generation (TG) of the nine gamers individually and the mean value at each time point. TG was generally not changed significantly during the gaming period. There was a trend towards an increase in ETP (*p* = 0.085), and the peak also increased slightly but non-significantly (*p* = 0.13), whereas there was no trend for Lagtime (*p* = 0.80) or time to peak (*p* = 0.65). 

**Figure 2:**
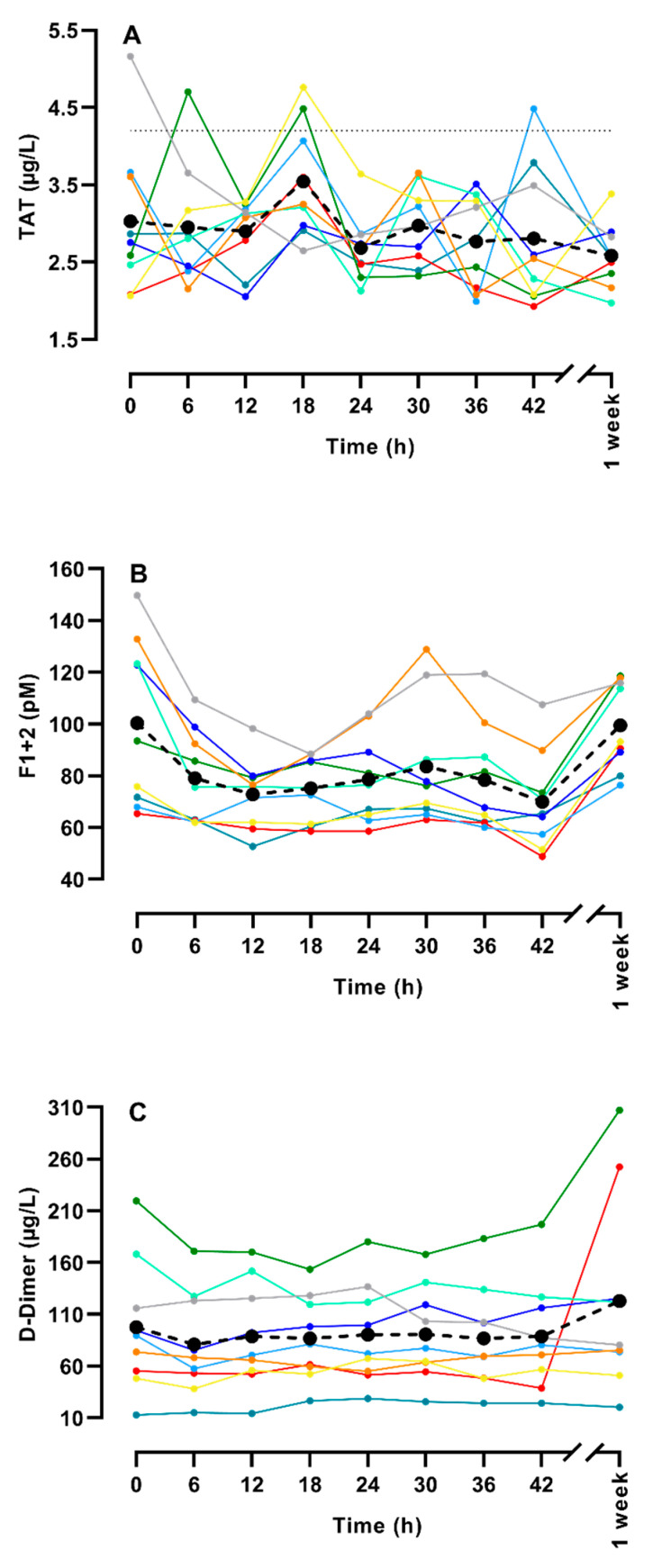
Measurement of thrombin antithrombin complex (TAT) (**A**), prothrombin fragment 1 + 2 (F1 + 2) (**B**), and D-Dimer (**C**) during a LAN party for 9 healthy gamers. Each colored line depicts one of the gamers, whereas the black dot (●) and the dashed black line depicts the mean of the nine gamers. The thin gray dotted line in figure A indicate the upper limit of the reference interval (4.2 µg/L).Please note that the values after one week are not included in the trend analysis—they are only included in the figure for comparison.

Figure 2 depicts the changes in TAT, F1 + 2, and D-dimer. A variation was seen for each participant, but almost all values were within the reference intervals, except for a few TAT results, and the mean level was relatively unchanged during the gaming period. There was no trend during the period for TAT (*p* = 0.30) or D-dimer (*p* = 0.67), whereas for F1 + 2, it actually decreased by 15% (*p* = 0.001). 

Details of the trend analyses are shown in Table 1. If the upper limit of the 95% confidence intervals (CIs) of the change during the session, i.e., maximal change, is inspected for clinical relevance, ETP and peak may increase somewhat (maximal values of 28 and 39% during 2 days, respectively), and a thrombogenic change cannot be excluded. However, this does not lead to a substantial change in the coagulation markers: F1 + 2 even decreases when using the upper limit of the 95% CI, and TAT and D-dimer would only increase negligibly.

General biochemical and hematological analyses were performed during the study [18]. P-albumin decreased almost 7% from the sample before the gaming period to the first sample after 6 h of gaming, but the level returned to the initial level during the 42 h period. B-hemoglobin had the same time course, with an initial decrease of 9%, which gradually normalized during the period. B-platelets decreased a little less but also increased by about 9% from the second sample to the end of the 42 h period. These changes were interpreted as changes in the hydration of the participants with initial overhydration. 

## 4. Discussion

The present study shows that TG and markers of coagulation did not demonstrate a significant thrombogenic change during 42 h of gaming with minimal physical activity and mainly unhealthy eating and drinking, i.e., we could not demonstrate a procoagulant response during a LAN party despite a rather long period of time with seated immobility. However, there was a trend towards an increase in ETP, but the markers of coagulation activation, TAT, and F1 + 2 did not show any increase. F1 + 2 actually decreased significantly, but this was in the opposite direction as expected, and, therefore, it did not indicate a procoagulant response.

Video gaming has become very popular in recent decades, and users may spend a long time in front of a computer. Immobility may constitute a risk of VTE, and several cases of VTE events in young gamers or other users of PCs have been described [4,5,6,7,8,9,10]. LAN parties may last 2–4 days, typically over a weekend, like the present one, with the participants playing on a computer most of the time, and generally, they engage in the overconsumption of food, mainly of items with a high level of fat or sugar. The present LAN party, including the food intake, etc., has been described recently [16]. Thus, a long period like this of immobility, an unhealthy diet, and limited sleep could potentially represent a health risk. 

Seated immobility is a risk factor for VTE. In a case–control study defining seated immobility as >8 h, West et al. found that this was a risk factor, increasing the risk by 1.8 times [19]. Later, the same group investigated a larger case–control population and found a risk factor of 2.8 after multivariate analysis [20], whereas, when using another control group in a later study, they found a smaller risk factor of 1.2 [21], but the risk was still significantly increased. In a database study including a large population, Suadicani et al. found that people in a job with prolonged sitting had an increased risk of VTE [22].

In the present study, however, we could not demonstrate significant procoagulant activation during two days of gaming using the coagulation test, thrombin generation, and the markers of procoagulant activity, TAT, F1 + 2, and D-dimer. As mentioned, stasis may lead to procoagulant changes in the endothelium and leucocytes, which potentially can result in the formation of TF and perhaps contact activation and the lowered activity of the coagulation inhibitors [15]. These are local effects, which will become diluted systemically, but should probably (although unknown) be detectable by sensitive tests, whereas other local effects of, e.g., the various blood cells [15,23] may not be detectable. Thrombin generation is a global test of the coagulation system. It has demonstrated a higher degree of coagulation activity in people with thromboses and lower activity in people with a bleeding tendency [24,25]. The Lagtime and ttPeak depend on the initiation of the coagulation system, i.e., mainly the concentration of the tissue factor and maybe Factor VII, as well as the potential activation of the contact activation system. The peak describes the highest level of thrombin after activation, and, thus, is an indication of “the power” of the system, whereas the ETP, the endogenous thrombin potential, describes the total amount of thrombin generated after activation. Therefore, ETP is the main indicator for the entire coagulation capacity. Lagtime and ttPeak showed an almost negligible change during the gaming period, whereas ETP (and to some degree, also the peak) showed a trend towards an increase during the event. We cannot exclude the possibility, therefore, that a larger study group would show a significant increase, i.e., we cannot exclude the possibility that immobilization may have an effect which, in combination with other potential concomitant risk factors and/or other local effects, could have a thrombogenic effect. In this study, however, we could not demonstrate any increase in the coagulation markers. TAT and F1 + 2 are formed during coagulation activity when antithrombin complexes with thrombin (TAT) and when prothrombin is activated by releasing a part of the molecule called F1 + 2, respectively. They are both markers of coagulation activity. TAT has a shorter half-life than F1 + 2, and, therefore, TAT is more likely to increase briefly after a shorter activation period, whereas F1 + 2, with a longer half-life, can show a prolonged increase in activity but with a smaller amplitude. D-dimer is a degradation product of fibrin, i.e., it is a marker of fibrin formation after coagulation activity and subsequent fibrinolytic activity. The half-life is longer than for F1 + 2, and it is, therefore, more likely to detect a longer-lasting increase in coagulation activity.

Procoagulant activity during LAN parties has not been measured before, but immobilization as a risk factor for VTE has been investigated in other partly comparable situations. Kabrhel et al. [26] investigated reasons for increases in D-dimer in hospital patients without VTE, and they found that immobility was a significant cause, but their definition of immobility was a prolonged or permanent inability to mobilize one or more limbs. It is well known that long-haul flights increase the risk of thrombosis [3,27], although factors other than sitting may be important (hypoxia, dehydration, etc.). Schreijer et al. [28] investigated the activation of the coagulation system in 71 healthy volunteers during an 8 h flight and compared it with the activation of the coagulation system in participants who participated in an 8 h movie session and 8 h of daily life. TAT increased more during the flight but neither F1 + 2 nor D-dimer changed significantly. Changes were most pronounced in women with *Factor V Leiden* and users of oral contraception, i.e., persons with other risk factors. Furthermore, they found that certain persons (independent of known risk factors) were more susceptible to increases. Schobersberger et al. [29] investigated 19 healthy volunteers during a 10 h ride on a bus interrupted by two overnight stays and a 10 h bus ride back. They found an insignificant increase in TAT and a significant increase in F1 + 2, whereas D-dimer did not change significantly. Ansari et al. [30] investigated 10 healthy adults during 8 h of prolonged sitting only interrupted by walking to the restroom. They found no increase in TAT, F1 + 2, or D-dimer. Thus, some studies have demonstrated increased markers of coagulation activity during this type of immobilization, but the results have not been consistent, and some studies indicate that additional factors may be involved in persons with signs of increased coagulation activity. In the present study, the participants had very little physical activity during a 42 h session with minimal sleep. Apparently, a short walk for food or visiting the restroom was sufficient to avoid the discernible activation of the coagulation system potentially caused by the immobilization.

The participants of LAN parties usually eat unhealthy food items containing rather high levels of fat. High-fat meals have been shown to increase the coagulation activity caused by an increased concentration of activated factor VII (FVIIa), which potentially could be a prothrombotic factor [31]. However, this effect was apparently limited in this study, since Lagtime and ttPeak did not decrease much, which would be the expected effect of an increased FVIIa.

Limitations. The group of participants was rather small, and definite conclusions cannot be drawn. We could only measure changes in samples from systemic blood and could not exclude the possibility that local prothrombotic changes may have taken place. The participants were not tested for the potential presence of procoagulant variants such as *Factor V Leiden*. However, if this was present in one or a few of the participants, this would probably result in more formation of the markers of coagulation activity, which did not increase. It would be a valuable extension of this study to carry out a similar investigation on gamers carrying some of these prothrombotic risk factors or having other prothrombotic risks as obesity.

## 5. Conclusions

These results do not indicate that the coagulation system is activated in healthy young males during 42 h of gaming with minimal physical activity. Thus, this study did not indicate that LAN parties implicate a risk of VTE in general, but we cannot exclude the possibility that the potential or capacity of the coagulation system increased, which, in combination with other risk factors, could be thrombogenic. Furthermore, we could only measure systemic effects and cannot exclude the possibility that local changes of importance for the risk of thrombosis occurred. 

## Figures and Tables

**Table 1:** Trend analysis for TG and coagulation markers showing the change per day, standard error (SE), and the maximal change during 2 days if the upper limits of the 95% confidence intervals (CIs) were used.

	Mean	Change/d	SE	*p*	95% CI-Interval	Max. Change during 2 Days
Lagtime (min)	8.605	−0.028	0.110	0.80	−0.243–0.187	−0.486
ETP (nM·min)	862.5	56.8	33.0	0.09	−7.8–121.5	243
Peak (nM)	74.0	6.3	4.2	0.13	−1.9–14.6	29.2
ttPeak (min)	14.86	−0.081	0.176	0.65	−0.43–0.27	−0.86
TAT	3.12	−0.15	0.14	0.30	−0.43–0.14	0.28
F1 + 2	89.4	−8.6	2.5	0.001	−13.5–(−3.7)	−7.4
D-dimer	90.1	−1.13	2.63	0.67	−6.3–4.0	8.0

## Data Availability

The original contributions and data are included in the article/Supplementary Materials; further inquiries can be directed to the corresponding author.

## Supplementary Materials

The following supporting information can be downloaded at: https://www.mdpi.com/article/10.3390/life14040525/s1, Supplementary Table S1: Characteristics of the participants.
