# Supplementary material for: Are Gamers Prone to eThrombosis during Long Gaming Sessions?
Source: Life (Basel). 2024 Apr 18;14(4):525. doi: 10.3390/life14040525 (PMC11051545; doi:10.3390/life14040525)
Supplement: Supplementary file 1 [file life-14-00525-s001.zip › life-2947989-supplementary.pdf]

Supplementary Table S1. Characteristics of the participants.

	Age	Height (cm)	Weight (kg)	BMI	WC (cm)
**Gamer 1**	24.5	180.5	66.5	20.4	77
**Gamer 2**	26.2	185	84	24.5	91
**Gamer 3**	29.5	189	73.7	20.6	82
**Gamer 4**	29.3	174.5	81.1	26.6	88
**Gamer 5**	28.3	185	95.3	27.8	102
**Gamer 6**	23.5	186	85.6	24.7	89
**Gamer 7**	23.9	180	94.1	29.0	99
**Gamer 8**	23.7	178.5	77.6	24.4	89
**Gamer 9**	23.3	186.5	87.5	25.2	82.5
**Median**	**24.5**	**185**	**84**	**24.7**	**89**
**Mean (±SD)**	**25.8 (±2.6)**	**182.8 (±4.6)**	**82.8 (±9.3)**	**24.8 (±2.9)**	**88.8 (±8.0)**
